# The Study on the Electrochemical Efficiency of Yttrium-Doped High-Entropy Perovskite Cathodes for Proton-Conducting Fuel Cells

**DOI:** 10.3390/ma18153569

**Published:** 2025-07-30

**Authors:** Bingxue Hou, Xintao Wang, Rui Tang, Wenqiang Zhong, Meiyu Zhu, Zanxiong Tan, Chengcheng Wang

**Affiliations:** 1 Aviation Engineering Institute, Civil Aviation Flight University of China, Guanghan 618037, China; bingxuehou@foxmail.com (B.H.);; 2 Industrial Training Center (School of Entrepreneurship and Innovation), Shen Zhen Polytechnic University, Shenzhen 518055, China; wangxintao@szpu.edu.cn (X.W.); zwq18211408611@163.com (W.Z.); kid1439@outlook.com (M.Z.);

**Keywords:** proton-conducting fuel cells (PCFCs), high-entropy perovskites, yttrium doping, oxygen reduction reaction (ORR), thermal expansion compatibility, electrochemical performance, intermediate-temperature solid oxide cell

## Abstract

The commercialization of proton-conducting fuel cells (PCFCs) is hindered by the limited electroactivity and durability of cathodes at intermediate temperatures ranging from 400 to 700 °C, a challenge exacerbated by an insufficient understanding of high-entropy perovskite (HEP) materials for oxygen reduction reaction (ORR) optimization. This study introduces an yttrium-doped HEP to address these limitations. A comparative analysis of Ce_0.2−x_Y_x_Ba_0.2_Sr_0.2_La_0.2_Ca_0.2_CoO_3−δ_ (x = 0, 0.2; designated as CBSLCC and YBSLCC) revealed that yttrium doping enhanced the ORR activity, reduced the thermal expansion coefficient (19.9 × 10^−6^ K^−1^, 30–900 °C), and improved the thermomechanical compatibility with the BaZr_0.1_Ce_0.7_Y_0.1_Yb_0.1_O_3−δ_ electrolytes. Electrochemical testing demonstrated a peak power density equal to 586 mW cm^−2^ at 700 °C, with a polarization resistance equaling 0.3 Ω cm^2^. Yttrium-induced lattice distortion promotes proton adsorption while suppressing detrimental Co spin-state transitions. These findings advance the development of durable, high-efficiency PCFC cathodes, offering immediate applications in clean energy systems, particularly for distributed power generation.

## 1. Introduction

Proton-conducting fuel cells (PCFCs) [1,2,3,4,5,6,7,8,9,10,11,12,13] are considered a promising technology for mitigating greenhouse gas emissions by efficiently converting chemical fuels, such as green hydrogen (H_2_), carbon monoxide (CO), and simple organics like formic acid (HCOOH), into electricity. Operating at intermediate temperatures (400–700 °C), PCFCs offer high energy conversion efficiency. However, their efficiency is hindered at lower temperatures due to the slow kinetics of the O_2_ reduction reaction (ORR), which significantly limits overall cell efficiency [14]. One of the key challenges in PCFC technology for intermediate-temperature operation is the synthesis of air electrodes that combine high catalytic activity with long-term structural stability [10,11,12,13,14,15].

La_0.8_Sr_0.2_MnO_3_ (LSCF), La_0.6_Sr_0.4_Co_0.2_Fe_0.8_O_3−δ_ (LSM), and La_0.6_Sr_0.4_CoO_3−δ_ (LSC) have been the focus of research for their application as O_2_ electrode materials in solid oxide fuel cells [16,17]. However, their performance in the context of PCFCs remains suboptimal. While numerous studies have highlighted the advantages of Co-based double perovskites, including fast ionic diffusion, enhanced surface catalytic activity, and favorable electrochemical properties, their practical application is limited. These materials typically exhibit a high thermal expansion coefficient, which can cause electrode delamination at elevated temperatures. Moreover, they suffer from instability in reducing or humid environments due to spin state transitions between Co^2+^ and Co^3+^.

In recent years, high-entropy perovskites (HEPs) [14,18,19,20,21,22,23,24]—perovskite oxides incorporating five or more different cations at the A- and/or B-sites—have attracted considerable attention. Their appeal lies in the ability to suppress cation diffusion within the lattice, therefore increasing structural stability. HEPs exhibit improved electronic properties, catalytic activity, electrochemical performance, and ionic transport, along with higher thermodynamic stability at elevated temperatures. These attributes, achieved in part by modulating thermal expansion behavior, make HEPs promising candidates for diverse applications, including water splitting, thermoelectric devices, electrochemical catalysis, and energy storage and conversion [25,26,27,28]. Therefore, the high-entropy strategy offers significant potential for enhancing structural tolerance and thermal compatibility, while simultaneously improving catalytic activity [29,30,31,32,33,34]. Chen Yu [1,2,14] conducted a systematic investigation into the development of high-entropy oxygen electrodes through A-site entropy engineering, synthesizing a series of Co-based perovskites such as Pr_0.2_Ba_0.2_Sr_0.2_La_0.2_Ca_0.2_CoO_3−d_ and Ce_0.2_Ba_0.2_Sr_0.2_La_0.2_Ca_0.2_CoO_3−d_ (CBSLCC). Their findings demonstrated outstanding cell performance and stability in PCFCs, offering a unique strategy for designing durable and active air electrodes suitable for efficient water splitting and O_2_ reduction. In a related study, Edith Bucher [35] explored the influence of yttrium (Y) on the mutual solubility of Ce^4+^ and Fe^3+^/Fe^4+^ within the Fe-rich and Ce-rich phases of BaCe_1−(x+z)_Fe_x_Y_z_O_3−δ_. The results revealed that only a small fraction of Y was incorporated into the electrolyte-type (Ce-rich) phase, and the resulting increase in proton uptake was limited, indicating the restricted addition of Y into the Ce-rich matrix.

Calcium ions (Ca^2+^) are commonly incorporated into the A-sites of perovskite oxides due to their chemical stability and cost-effectiveness [36,37,38]. Moreover, Ca^2+^ contributes to enhanced resistance against CO_2_ poisoning and steam-induced degradation [39]. The ionic radii of Ce^3+^ (1.15 Å), Ce^4+^ (1.01 Å), and Y^3+^ (0.90 Å) were obtained from Shannon’s ionic radii database, accounting for their respective valence states and a coordination number of six. The significant size mismatch between Ce and Y at the A-site of the ABO_3_ perovskite structure is considered a primary factor driving the phase separation of the precursors into distinct structural domains. This work addresses these gaps by designing Ce_0.2−x_Y_x_Ba_0.2_Sr_0.2_La_0.2_Ca_0.2_CoO_3−δ_ (x = 0, 0.2) cathodes to elucidate yttrium’s role in enhancing ORR activity and thermal compatibility, quantifying proton uptake and electrochemical performance under operating conditions, and establishing a scalable HEP framework for next-generation PCFCs.

## 2. Materials and Methods

### 2.1. Materials Preparation

Ce_0.2−x_Y_x_Ba_0.2_Sr_0.2_La_0.2_Ca_0.2_CoO_3−δ_ (x = 0, 0.2) (CYBSLCC) powder was synthesized by utilizing the ethylenediaminetetraacetic acid (EDTA)-citrate technique, outlined in a prior paper [29,40]. All chemical reagents, including Ce(NO_3_)_2_, Y(NO_3_)_2_, Ba(NO_3_)_2_, Sr(NO_3_)_2_, La(NO_3_)_3_·6H_2_O, Ca(NO_3_)_2_, Co(NO_3_)_2_·6H_2_O, citric acid (CA), EDTA, and aqueous ammonia (NH_3_·H_2_O), were provided by Sigma Corp. The synthesis was initiated by dissolving the appropriate stoichiometric amounts of metal nitrates in distilled water to obtain a clear mixture. The CA solution was then introduced as a chelating agent, followed by the addition of EDTA pre-dissolved in NH_3_·H_2_O to ensure complete solubilization and to adjust the pH to approximately 10. The molar ratio of the total metal cations to EDTA and CA was set to 1:1:1.5. The resultant homogeneous solution was continuously stirred for 4 h at a temperature equal to 80 °C to promote gelation. The prepared gel was subsequently calcined in air at 600 °C to decompose organic components and nitrate residues. After that, the resulting powder was subjected to high-temperature treatment for 4 h at 1000 °C to achieve crystallization of the desired perovskite phase.

Commercially fabricated PCFC button half-cells (NiO-BZCYYb|BZCYYb) with a diameter equal to 16 mm were sourced from a local supplier for full-cell assembly and evaluation. Here, BZCYYb refers to the composition BaZr_0.1_Ce_0.7_Y_0.1_Yb_0.1_O_3−δ_, while NiO–BZCYYb denotes a 50:50 weight ratio mixture of NiO and BZCYYb. Cathode inks for Ce_0.2−x_Y_x_Ba_0.2_Sr_0.2_La_0.2_Ca_0.2_CoO_3−δ_ (x = 0, 0.2) compositions were prepared using a binder system comprising 97 wt% terpineol and 3 wt% ethyl cellulose. The binder mixture was homogenized at 80 °C to form a uniform medium. The cathode powders were then dispersed into this binder solution in equal proportions (1:1 by mass) to form slurries. These slurries were manually applied via brush-coating onto the electrolyte surfaces of the half-cells. The coated samples were subsequently sintered in air for 3 h at 1050 °C to produce porous cathode layers with a mean thickness of around 20 μm and a geometric area equal to 0.5 cm^2^.

### 2.2. Characterization Techniques

A D8 Advance X-Ray diffraction (XRD) system from Bruker was utilized to ascertain the structural formation of Ce_0.2−x_Y_x_Ba_0.2_Sr_0.2_La_0.2_Ca_0.2_CoO_3−δ_ (x = 0, 0.2) powders and chemical compatibility of three kinds of powders with the BZCYYb electrolyte from 10° to 80° (2*θ*). The morphology of powders was further examined using a Titan G2 60-300transmission electron microscope (TEM) (Thermo Fisher Scientific, Hillsboro, OR, USA), operated at an accelerating voltage equal to 200 kV. Thermal expansion behavior was assessed using a dilatometer (TA Instruments, DIL802, New Castle, DE, USA). The measurements were conducted in air from 30 to 900 °C at a ramp rate equal to 5 °C min^−1^, employing sintered pellets as specimens. The preparation of Ce_0.2−x_Y_x_Ba_0.2_Sr_0.2_La_0.2_Ca_0.2_CoO_3−δ_ (x = 0, 0.2) pellets involved initial weighing of the powders, followed by molding into circular shapes and pressing in a hydraulic press. Subsequently, the pellets were treated at a high temperature value equal to 1400 °C for a duration equal to 4 h. The surface chemical distribution of the Ce_0.2−x_Y_x_Ba_0.2_Sr_0.2_La_0.2_Ca_0.2_CoO_3−δ_ (x = 0.2) powders was determined using an ESCALAB 250Xi X-ray photoelectron spectroscopy (XPS) setup from Thermo Fisher Scientific.

The microstructural features of the cells prior to and following electrochemical testing were analyzed using a NEON 40ESB scanning electron microscopy (SEM) (Carl Zeiss AG, Oberkochen, Germany). The electrochemical efficiency of single cells based on Ce_0_._2−x_Y_x_Ba_0_._2_Sr_0_._2_La_0_._2_Ca_0_._2_CoO_3−δ_ (x = 0, 0.2) was evaluated under ambient air conditions, with humidified H_2_ (100 mL·min^−1^) provided as the fuel at 700 °C. Current–voltage (I–V) tests were conducted using a Solartron 1260 system (Solartron Analytical, Farnborough, UK). Electrochemical impedance spectroscopy (EIS) was carried out at 700 °C inside air within a frequency range extending from 10^6^ to 10^−1^ Hz with an applied AC amplitude equal to 10 mV. Impedance data were analyzed and fitted using Zivew 2 software.

## 3. Results and Discussion

### 3.1. Morphological and Structural Analysis

**Figure 1:**
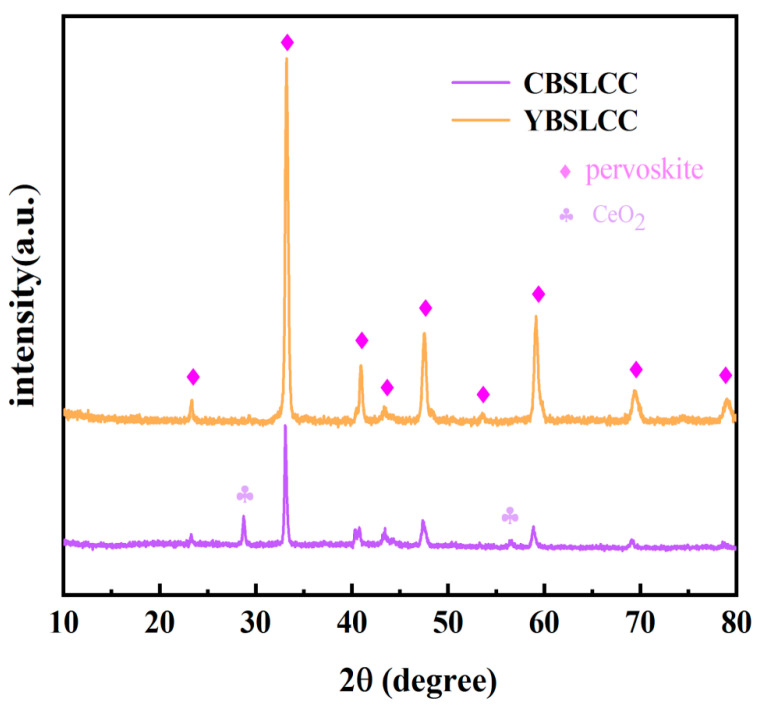
The XRD profiles obtained for Ce_0.2−x_Y_x_Ba_0.2_Sr_0.2_La_0.2_Ca_0.2_CoO_3−δ_ (x = 0, 0.2) powders calcined for 3 h at 1050 °C in air.

**Figure 2:**
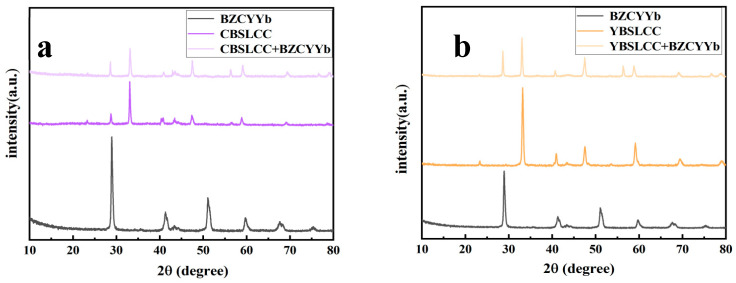
XRD patterns of chemical compatibility of Ce_0.2−x_Y_x_Ba_0.2_Sr_0.2_La_0.2_Ca_0.2_CoO_3−δ_ (x = 0, 0.2) powders with the BZCYYb electrolyte powders calcined for 10 h at a temperature equal to 1050 °C in air. (**a**) x = 0; (**b**) x = 0.2.

The XRD profiles obtained for the Ce_0.2−x_Y_x_Ba_0.2_Sr_0.2_La_0.2_Ca_0.2_CoO_3−δ_ (x = 0, 0.2) powders calcined for 4 h at a temperature equal to 1000 °C in an ambient air environment are presented in Figure 1. It is evident that CBSLCC (x = 0.2) mainly comprises three phases, including the Ce_0.2−x_Y_x_Ba_0.2_Sr_0.2_La_0.2_Ca_0.2_CoO_3−δ_(CD-CBSLCC) perovskite phase (PDF:48-0121), CeO_2_ (PDF:89-8436), and a minor fraction of the La_0.7_Sr_0.3_CoO_3−d_ (LSC) perovskite phase (PDF:51-0405). In the case of YBSLCC (x = 0), the XRD analysis confirmed the presence of a predominant perovskite phase corresponding to Ce_0.2−x_Y_x_Ba_0.2_Sr_0.2_La_0.2_Ca_0.2_CoO_3−δ_ (CD-YBSLCC; PDF:48-0121) and a minor amount of La_0.7_Sr_0.3_CoO_3−d_ (LSC; PDF:51-0405). To evaluate the configurational entropy and structural suitability, the mixing entropy (ΔS_mix_) and Goldschmidt tolerance factor (t) were calculated for both CBSLCC and YBSLCC compositions. The ΔS_mix_ values exceeded −1.5R in both cases, qualifying them as high-entropy materials. Structural stability was further assessed using the Goldschmidt tolerance factor, which is defined by t=(rA+rO)/√2(rB + rO), where rO, rA, and rB represent the average ionic radii of the O_2_ anions (O^2−^), along with the A- and B-site cations, respectively. The t-values calculated for CBSLCC and YBSLCC were 0.87 and 0.80, respectively, both within the generally accepted stability range of 0.78 to 1.05, although values below 1 suggest a deviation from the ideal cubic perovskite structure. Figure 2 presents a comparative XRD analysis of BZCYYb, CBSLCC, YBSLCC, and a 1:1 (by mass) mixture of CBSLCC and YBSLCC with BZCYYb, calcined for 100 h at a temperature equal to 1100 °C. The absence of secondary phases in the mixed samples confirms the excellent chemical compatibility among CBSLCC, YBSLCC, and BZCYYb.

**Figure 3:**
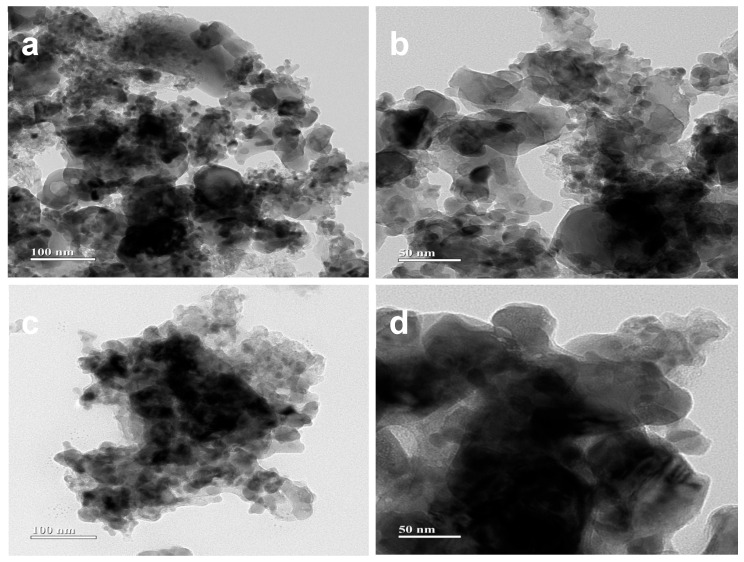
HRTEM analysis of Ce_0.2−x_Y_x_Ba_0.2_Sr_0.2_La_0.2_Ca_0.2_CoO_3−δ_ (x = 0,0.2) ((**a**,**b**), x = 0; (**c**,**d**), x = 0.2).

TEM micrographs of CBSLCC (Figure 3a,b) and YBSLCC (Figure 3c,d) showed well-dispersed, quasi-spherical nanoparticles with minimal agglomeration, likely resulting from weak van der Waals interactions. Particle size distribution analysis showed a narrow size range of 15–25 nm (mean diameter: 20 ± 3 nm). These observations confirm the high crystallinity and phase purity of the synthesized nanomaterials. Elemental mapping of individual nanoparticles confirmed the presence of Ce, Ba, Sr, La, Ca, and Co, with the homogeneous distribution of A-site dopants in the CBSLCC composition, indicating the absence of cation segregation.

### 3.2. Thermal Property and Elemental Analysis

**Figure 4:**
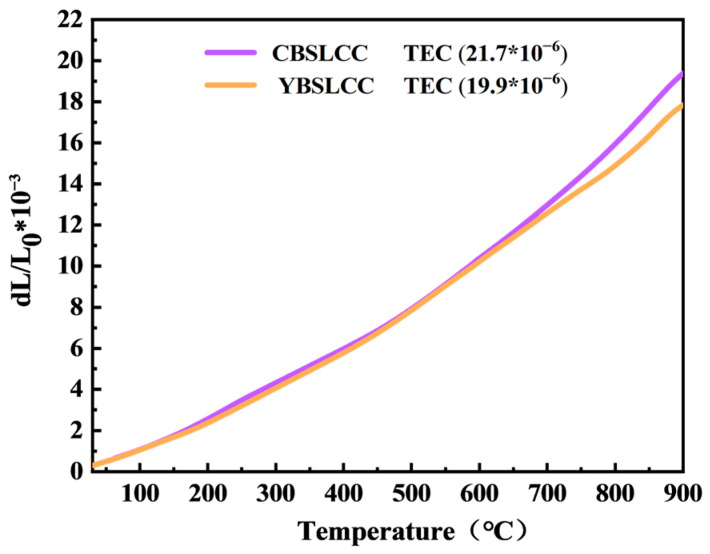
Thermal expansion coefficient (TEC) curve of the as-prepared Ce_0.2−x_Y_x_Ba_0.2_Sr_0.2_La_0.2_Ca_0.2_CoO_3−δ_ (x = 0, 0.2) vs. temperature (30–900 °C).

The thermal expansion coefficient (TEC) of the O_2_ electrode is widely recognized as a pivotal parameter for its application in PCFCs. The thermal expansion behavior of the synthesized CBSLCC and YBSLCC samples within the temperature range extending from 30 to 900 °C is illustrated in Figure 4. The average TEC of CBSLCC was determined to be 21.7 × 10^−6^ K^−1^, consistent with previously reported values for similar cobalt-based perovskites. However, YBSLCC exhibited a lower average TEC of 19.9 × 10^−6^ K^−1^ within the same temperature range, suggesting improved thermomechanical compatibility with the BZCYYb electrolyte. The reduction in TEC upon Y incorporation into the ABO_3_ lattice is likely attributed to modifications of electrostatic interactions and interatomic distances, driven by differences in electronegativity and ionic radii among the constituent A-site cations. Further, the slightly reduced oxygen loss and lower TEC observed for YBSLCC at higher temperature values highlight the benefits of the A-site high-entropy design in increasing thermal stability and interfacial compatibility.

**Figure 5:**
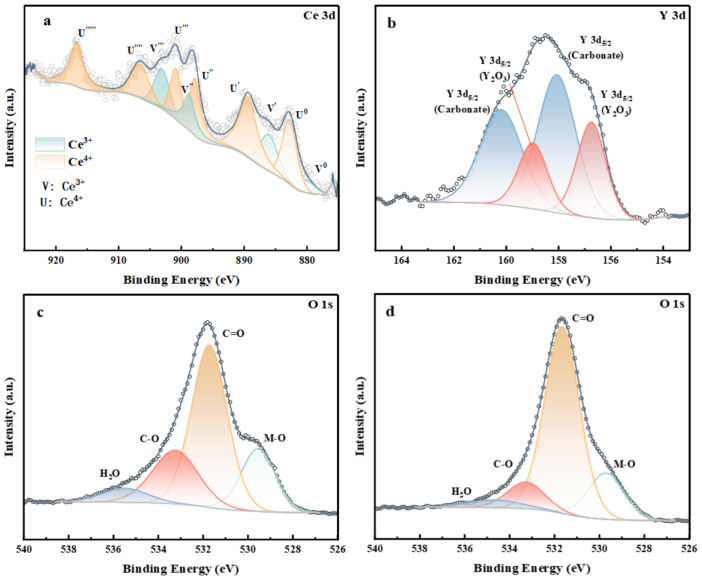
Deconvoluted XPS test results for the surface of the chosen Ce3d (**a**), Y3d (**b**), O1s ((**c**): x = 0), O1s ((**d**):x = 0.2) of as-prepared Ce_0.2−x_Y_x_Ba_0.2_Sr_0.2_La_0.2_Ca_0.2_CoO_3−δ_ (x = 0, 0.2) powders.

The valence states and surface chemical composition of the doped elements within the CBSLCC and YBSLCC powders were determined via XPS analysis. Figure 5 displays the XPS survey and high-resolution spectra, including the deconvoluted Ce 3d (Figure 5a), Y 3d (Figure 5b), and O 1s (Figure 5c,d) core levels for both materials. In the Ce 3d spectrum of CBSLCC (Figure 5a), the dominant peaks at binding energies equal to 882.76 and 889.4 eV are attributed to Ce^4+^ species within the perovskite lattice. Additional signals at 886.08 and 903.08 eV, corresponding to Ce^3+^, were also observed, indicating a mixed-valence state. The coexistence of Ce^4+^ and Ce^3+^ without clear surface-bulk differentiation suggests a uniform distribution of cerium oxidation states throughout the structure, which contrasts with previous reports showing distinct Ce surface species. The high-resolution Y 3d spectrum of YBSLCC (Figure 5b) reveals primary peaks at 158.06 eV and 160.19 eV, consistent with Y^3+^ within the perovskite framework. Deconvolution further revealed additional peaks at 156.74 eV and 158.97 eV, indicating the presence of Y_2_O_3_ and implying a heterogeneous distribution of yttrium, with both bulk and surface species. The O 1s spectra for both samples (Figure 5c,d) show multiple contributions. In addition to typical surface-adsorbed carbon species (C=O and C–O), a significant peak attributed to metal–oxygen (M–O) bonding was identified, confirming the formation of a robust perovskite lattice structure.

### 3.3. Micro-Structural Characterization of Single Cells Before Electrochemical Tests

**Figure 6:**
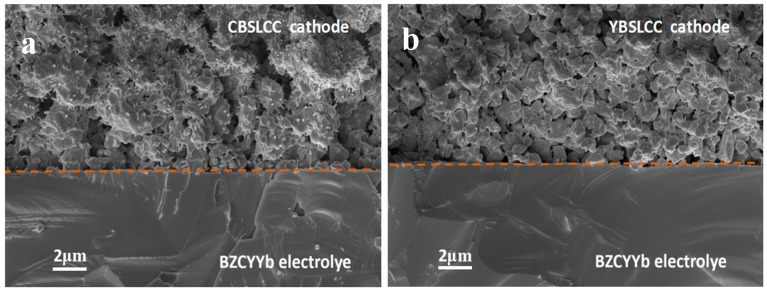
The cross-sectional SEM micrographs obtained for the anode-supported single cell with Ce_0.2−x_Y_x_Ba_0.2_Sr_0.2_La_0.2_Ca_0.2_CoO_3−δ_ (x = 0, 0.2) electrodes/BZCYYb electrolytes before the electrochemical tests. (**a**) x = 0; (**b**) x = 0.2. Note: dotted line was used to differentiate the cathode and electrolyte.

Figure 6a,b display the cross-sectional SEM images of CBSLCC- and YBSLCC-based single cells before the electrochemical evaluation. Both cathodes exhibited highly porous microstructures, essential for efficient gas diffusion and the development of the triple-phase boundaries necessary for optimal performance in protonic ceramic fuel cells (PCFCs). The GDC electrolyte layers were dense and free of cracks, indicating excellent mechanical integrity and reliable ionic conductivity at elevated operating temperatures. The interfaces between the cathodes and electrolytes were well-adhered, with no evidence of delamination or interfacial voids, confirming good compatibility and fabrication quality. A comparative microstructural analysis revealed an evident difference in particle size distribution: the YBSLCC cathode (Figure 6b) showed a more refined and uniform microstructure, with particle sizes ranging from ~0.5 to 1.0 μm, while the CBSLCC cathode (Figure 6a) exhibited larger particles in the range of ~1.0 to 2.0 μm. The finer particle size and more homogeneous morphology of YBSLCC are expected to enhance catalytic activity by increasing the electrochemically active surface area. Overall, both cells demonstrated microstructural features well-aligned with the requirements for high-performance solid-state electrochemical devices, offering a favorable balance between cathode porosity and electrolyte densification.

### 3.4. Electrochemical Efficiency of Single Cells

**Figure 7:**
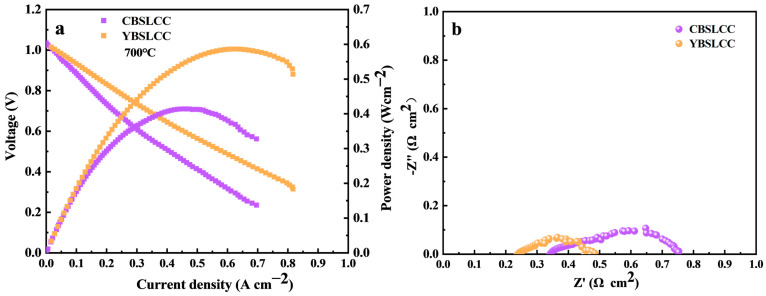
I-V curves (**a**) and impedance curves (**b**) of Ce_0.2−x_Y_x_Ba_0.2_Sr_0.2_La_0.2_Ca_0.2_CoO_3−δ_ (x = 0, 0.2) single cells measured at 700 °C under the testing condition.

The electrochemical efficiency of single cells incorporating CBSLCC and YBSLCC as cathode materials was assessed under fuel cell operating at conditions of 700 °C using humidified H_2_ (3% H_2_O) as the fuel. Both cells exhibited characteristic current–voltage–power (I–V–P) profiles, as shown in Figure 7a. The CBSLCC-based cell achieved a peak power density (P_max_) equal to 400 mWcm^−2^, while the YBSLCC-based cell delivered a significantly higher P_max_ of 586 mWcm^−2^ under identical conditions. EIS data, presented in Figure 7b, revealed polarization resistances equal to 0.4 Ω·cm^2^ for CBSLCC and 0.3 Ω·cm^2^ for YBSLCC at an open-circuit voltage (OCV), further confirming the enhanced performance of the YBSLCC cathode. Although the P_max_ achieved with the YBSLCC electrode does not surpass the highest values reported in the literature [1], the improvement over CBSLCC is evident and warrants further examination. The enhanced performance associated with Y-doping can be ascribed to several factors. Substituting Ce^4+^ by Y^3+^ increased the O_2_ vacancy concentration and enhanced the basicity, particularly in Ce-rich regions, which are critical for proton conduction. However, the mismatch in ionic radii between Y^3+^ (0.90 Å) and Ce^4+^ (0.87 Å) introduces local lattice strain within the Ce-rich phase. The partial incorporation of Y^3+^ into these regions may help relieve this strain through cation intermixing, but it can also suppress proton uptake due to decreased oxygen vacancy mobility. This complex interplay likely governs the improved electrochemical response observed for YBSLCC.

### 3.5. Micro-Structural Characterization of Single Cells After Tests

**Figure 8:**
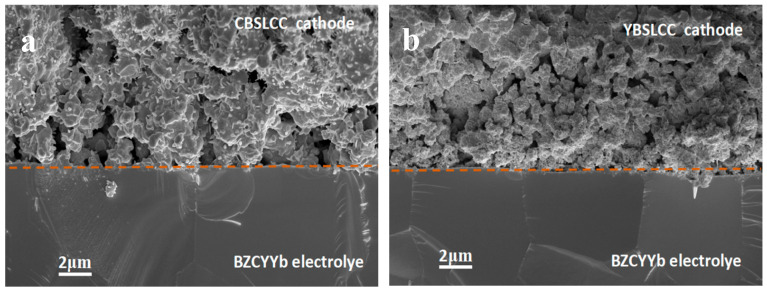
Cross-section SEM micrographs of anode-supported single cell with Ce_0.2−x_Y_x_Ba_0.2_Sr_0.2_La_0.2_Ca_0.2_CoO_3−δ_ (x = 0,0.2) electrodes/BZCYYb electrolytes after the electrochemical tests. (**a**) x = 0; (**b**) x = 0.2. Note: dotted line was used to differentiate the cathode and electrolyte.

Cross-sectional SEM analysis of the two single cells following electrochemical testing (Figure 8a,b) confirms that the structural integrity of both anode and cathode layers was preserved, with well-maintained porous architectures essential for facilitating electrochemical reactions and gas diffusion. The BZCYYb electrolyte remained dense and defect-free, providing reliable ionic conductivity and mechanical durability during operation. The interfaces between the cathode layers and the electrolyte remained intact, showing no evidence of delamination, highlighting the strong interfacial bonding achieved through the co-sintering process. Of particular interest, the YBSLCC-based cathode (Figure 8b) exhibited a more refined microstructure with smaller, uniformly distributed particles averaging ~0.3–0.8 µm, in contrast to the larger grains (~1–2 µm) observed in the CBSLCC electrode (Figure 8a). This microstructural refinement in YBSLCC is believed to improve catalytic efficiency by increasing the density of active sites and triple-phase boundaries, thus facilitating ORR kinetics, consistent with its improved electrochemical performance.

## 4. Conclusions

This study demonstrates that yttrium doping in high-entropy perovskite cathodes (YBSLCC) significantly improves PCFC performance via three key mechanisms: yttrium increases the oxygen vacancy concentration and lattice distortion, attaining a peak power density equal to 586 mWcm^−2^ at 700 °C, 46% higher than undoped CBSLCC. The reduced thermal expansion coefficient (19.9 × 10^−6^ K^−1)^ mitigates interfacial delamination with BZCYYb electrolytes, critical for long-term stability. Y^3+^ incorporation boosts proton uptake while stabilizing Co^3+^, suppressing detrimental spin-state transitions. These results provide a blueprint for HEP cathodes in intermediate-temperature PCFCs, with immediate relevance to industries requiring efficient, durable energy systems, such as auxiliary power units and decentralized power grids. Future work should explore combinatorial doping strategies to further optimize proton conductivity and CO_2_ tolerance.

## Data Availability

The original contributions presented in this study are included in the article. Further inquiries can be directed to the corresponding author.

## Abbreviations

The following abbreviations are used in this manuscript:

PCFCs	Proton-conducting fuel cells
HEP	High-entropy perovskite
ORR	Oxygen reduction reaction
CO	Carbon monoxide
EDTA	Ethylenediaminetetraacetic acid
XRD	X-Ray diffraction
SEM	Scanning electron microscopy
EIS	Electrochemical impedance spectroscopy
TEC	Thermal expansion coefficient
OCV	Open-circuit voltage
